# Functional brain connectivity during exposure to the scale and color of interior built environments

**DOI:** 10.1002/hbm.26061

**Published:** 2022-09-02

**Authors:** Isabella S. Bower, Aron T. Hill, Peter G. Enticott

**Affiliations:** ^1^ Cognitive Neuroscience Unit, School of Psychology, Faculty of Health Deakin University Geelong Victoria Australia; ^2^ School of Architecture and Built Environment, Faculty of Science, Engineering and Built Environment Deakin University Geelong Victoria Australia

**Keywords:** brain connectivity, cortical oscillations, electroencephalography (EEG), environmental psychology, immersive virtual reality, spatial cognition, visual perception

## Abstract

Understanding brain activity linked to built environment exposure is important, as it may affect underlying cognitive, perceptual, and emotional processes, which have a critical influence in our daily life. As our time spent inside buildings is rising, and mental health problems have become more prevalent, it is important we investigate how design characteristics of the built environment impact brain function. In this study, we utilized electroencephalography to understand whether the design elements of scale and color of interior built environments modulate functional brain connectivity (i.e., brain network communication). Using a Cave Automatic Virtual Environment, while controlling indoor environmental quality responsible for physiological comfort, healthy adult participants aged 18–55 years (66 for scale, subset of 18 for color), were exposed to context‐neutral indoor room scenes presented for two‐minutes each. Our results show that both enlarging and reducing scale enhanced theta connectivity across the left temporoparietal region and right frontal region. We also found when reducing the built environment scale, there was a network exhibiting greater high‐gamma connectivity, over the right frontoparietal region. For color, the condition (blue) contrasted to our achromatic control (white) increased theta connectivity in the frontal hemispheres. These findings identify a link between theta and gamma oscillations during exposure to the scale and color of the built environment, showing that design characteristics of the built environment could affect our cognitive processes and mental health. This suggests that, through the design of buildings, we may be able to mediate performance and health outcomes, which could lead to major health and economic benefits for society.

## Graphical abstract

There is evidence emerging of the psychological impact of the built environment, but not the accompanying brain‐based evidence. As built environment exposure may have implications on our cognitive, attentional, perceptual, and emotional functioning, it is critical we understand and measure this impact. Here, we use a rigorous interdisciplinary approach using electroencephalography, virtual reality, and indoor environmental quality monitoring to understand if design elements of an interior room impact functional connectivity networks. The study confirmed that brain network activity is impacted by scale and color, demonstrating this approach is a viable method for investigating the health implications for time spent within built environments. This research raises the prospect that neuroscience methods could contribute an evidence base to guide building design and evaluation.

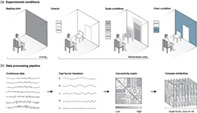

## 1 INTRODUCTION

It is estimated we spend approximately 90% of our time inside buildings (Klepeis et al., 2001). During the COVID‐19 pandemic this time increased, and our exposure to a variety of built environments such as workplaces, schools, community, and commercial venues diminished as strict restrictions, regulations, and lockdown protocols were imposed to limit socially driven virus transmission occurrences. As a result, much of the population experienced reduced environmental enrichment in the form of in‐person social interactions and varied physical surrounds. Environmental enrichment refers to the environmental conditions facilitating enhanced sensory, cognitive, and motor stimulation (Nithianantharajah & Hannan, 2006). Studies have begun to reveal that this lack of enrichment has had adverse effects on mental health (Amerio et al., 2021; Stieger et al., 2021), with confined quarantine environments found to be most mentally detrimental (Brooks et al., 2020) and akin to the negative psychological effects resultant from sensory deprivation in the physical and social environment (Mackes et al., 2020). However, despite the greater time spent indoors, there is little research investigating the impact of interior built environments on brain activity (Bower et al., 2019; Moore et al., 2018). As mental health issues in society continue to rise, and indoor environments play a key role in our lifestyles, it is critical we understand whether the design of built environments impact our brain function, and if so, recognize the implications this might have on our cognitive, attentional, perceptual, and emotional functioning.

Understanding the impact of built environment exposure on brain activity is inherently complex due to the vast quantity and variety of spaces we visit or occupy, and our pre‐existing associations and memories linked to built environments, which can confound the results. Often, we also hold goal orientated behaviors when immersed in the built environment, resulting in spatial priming, where we use a memory from prior perceptual experience to optimize our focus and behavior and increase task efficiency (Sanocki & Epstein, 1997). While studies have broadly investigated differences in neural processing between naturalistic and urban environments (Norwood et al., 2019; Roe et al., 2013), external environments are subject to many confounding variables such as atmospheric conditions (weather and climate), how they are presented (still images, virtual reality [VR], or field‐based exposure), how they are constructed (complexity and materiality) and how they are composed (viewpoint and perspective). Inside buildings, the overarching context of the building (residential, commercial, educational, and healthcare); comfort properties (thermal comfort, indoor air quality, lighting, and acoustics); contents (furniture, fixtures, equipment, and other physical artifacts); and occupants (social distances, interactions, and relationships) also increase the variation in what we are exposed to. Emerging research is investigating whether we can measure the impact of built environment design characteristics on brain activity. Some studies have taken a macro approach by determining broad descriptors of complex built environments, such as “fascination,” “coherence,” and “hominess” (Coburn et al., 2020), while others have employed an incremental method by breaking down the built environment into fundamental physical design elements, such as “geometry,” “texture,” “scale,” and “color,” to understand them at the microlevel before building complexity into the experimental design (Bower et al., 2022a, 2022b).

Electroencephalography (EEG) is a noninvasive and temporally precise method for analyzing neural activity during different tasks and conditions, such as in response to visual stimuli. While previous studies using EEG to investigate the impact of the built environment on brain activity have analyzed spectral power (Llinares et al., 2021; Vecchiato et al., 2015), and event‐related potentials (Djebbara et al., 2019), no work to date has examined functional connectivity. Originating from neuroimaging, functional connectivity describes the temporal correlation between two distinct neurophysiological events, to understand whether there is a statistical relationship between the activity recorded at different spatial regions within the brain (Friston, 1994). Functional connectivity can therefore provide an indication of the pattern and strength of communications between different brain regions (i.e., brain circuits or networks), both local (short‐range) and global (long‐range). Functional connectivity studies have predominantly used data from neuroimaging techniques, such as functional magnetic resonance imaging (fMRI); however, a growing body of work has begun using EEG. The use of EEG enables greater ecological validity for participant experience as they are not restricted to the supine position in a confined space when undergoing a scan. Current work investigating functional connectivity in fMRI data have contrasted vastly different stimuli, with a focus on understanding the difference between natural versus human‐made built environment scenes (Kühn et al., 2021). Critically, this work found that functional connectivity was able to detect changes in neural activity that was undetected in participant self‐report. However, there are constraints on ecological validity in the testing environment (clinical/confined MRI setting) and stimuli (photographic scene).

In this study, we investigated whether functional connectivity could be used to elucidate the impact of the built environment on our brain activity, in the context of scale and color. We selected these two variables as they can be perceived without the need of additional visual properties or sensory processes. This limits the complexity expected in neural processing, as the addition of other properties (i.e., luminance and/or texture to perceive form) or additional sensory inputs (i.e., touch and/or movement to perceive texture) are found to involve different processing mechanisms (Whitaker et al., 2008). Understanding the implications of scale applied to the built environment is not new, with studies that have investigated room size on behavioral measures (Evans et al., 1996; Wolfe, 1975), self‐report (Sander et al., 2019), and more recently, brain processing of distances when exposed to scale categories varying in magnitude from room to continent (Peer et al., 2019). However, prior studies often do not distinguish between the physical and social environment, making it difficult to understand whether there is a direct effect of the physical environment. We are also at an early stage of understanding the impact of color (Elliot, 2015), with studies often presenting methodological problems (Wilms & Oberfeld, 2018). Nevertheless, there is a growing body of emerging work investigating the relationship between built environment color and self‐reported mood (Lipson‐Smith et al., 2021), attention, and memory (Llinares et al., 2021).

**FIGURE 1:**
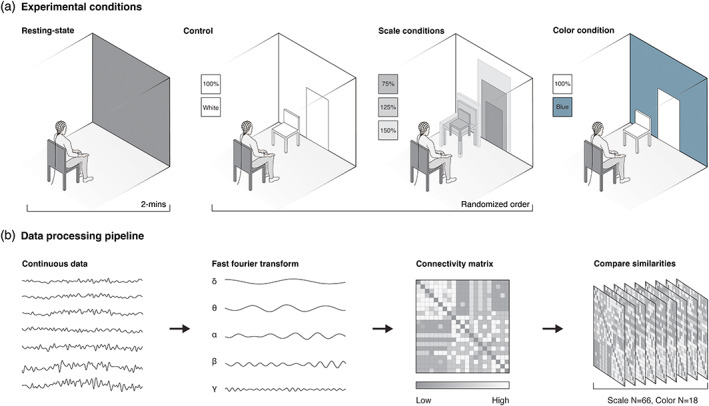
Experimental conditions and data processing pipeline schematic. (a) Isometric schematic of the experimental conditions. (b) Summary of the analysis pipeline transforming electroencephalography (EEG) data for functional brain connectivity analysis. Diagrams are representative, not drawn to exact scale/numbers.

Here, we test a rigorously controlled method to measure EEG functional connectivity during exposure to scale and color of interior built environment scenes. The use VR and indoor environmental quality monitoring, makes this a novel and robust approach to understanding the connection between the built environment and neuroscience. This study follows earlier work where we investigated changes in self‐report, power spectral analysis, and physiological indictors to scale and color of the built environment (Bower et al., 2022a, 2022b). A Cave Automatic Virtual Environment (CAVE) was used to expose participants to indoor built environment scenes, a cost‐effective and controlled technique which enables greater sensorimotor integration than VR headsets (Kalantari et al., 2021; Sanchez‐Vives & Slater, 2005). The virtual built environment scene we modulated in scale and color was a room which included a closed door and a chair, providing participants visual cues to determine height, width, and surface depth (Brouwer et al., 2005). These items were selected to be most neutral of the environmental context, with their dimensions modeled from the local regulatory standards for a controlled approach. Participants were presented with an eyes‐open resting‐state, followed by four randomized scale scenes, where the room, door, and chair changed in scale (75%, 100%, 125%, and 150%). Finally, a color scene (blue) was presented at the end. All scenes lasted two‐minutes, between which the resting‐state was displayed while a self‐report task was completed (not reported in this manuscript). Due to the exploratory nature of this study, we took a data‐driven approach without specific predictions. We ran comparisons of connectivity for each of the canonical EEG frequency bands averaged across participants for each condition, using *t* tests to compare between conditions (Figure 1).

## 2 MATERIALS AND METHODS

### 2.1 Participants

66 healthy adult volunteers (31 females, age = 34.9 ± 11.3 years) participated in the scale component of this study, while 18 participants participated in both the scale and color component (8 females, age = 34.5 ± 9.87 years). All participants provided written, informed consent approved by the Deakin University Human Research Ethics Committee. No participants reported prior diagnosed psychiatric, neurological, or neurodevelopmental conditions, or previous training or work experience in built environment design. A healthy adult sample was selected due to the experimental nature of the study and to reduce confounding variables. All participants were able to speak and read English and had normal or corrected to normal visual acuity.

### 2.2 Experimental design

An eyes open resting‐state was incorporated at the start and between each of the scenes for two‐minutes. Participants were seated comfortably in the center of the CAVE and instructed to keep their eyes‐open and pay attention to the scene they would be presented with. They were then exposed to four randomized (100 subjects, 1 block, seed 28107) achromatic built environment scenes, and one chromatic color condition at the end. Each scene was presented for two‐minutes, before the CAVE returned to the resting‐state display. This study used an eyes‐open resting‐state design due to the visual nature of the stimuli. Participants were exposed to varying built environment scenes using a standard sitting posture which is not uncommon for the type of study or real‐world behavior within the built environment. This helped minimize differences in head height and reduced movement artifact in recordings; however, it is worth noting subject positioning can alter the neurophysiological activity (Puce & Hämäläinen, 2017). We elected not to involve task‐based measures as these would distract from the scene exposure, and that it is not uncommon to spend time within buildings without necessarily performing a task or planning an action. However, we recognize this is an under examined area of research, warranting future exploration. A period of two‐minutes per condition was selected to ensure sufficient EEG data to epoch for stability in connectivity analysis (van Diessen et al., 2015), while avoiding habituation from the experimental conditions and nature of the scenes that could confound the results (O'Gorman, 1977).

The virtual built environment scene we modulated in scale was a room which included a closed door and a chair, providing participants visual cues to determine height, width, and surface depth (Brouwer et al., 2005). These items were selected to be most neutral of the environmental context, with their dimensions modelled from the local regulatory standards for a controlled approach. The scale scenes included a control scale scene (100%), modeled to the dimensions of Standards Australia for a residential internal door (Standards Australia, 2017), and three‐scale conditions where the scene was reduced in scale: small (75%), or increased: large (125%), and extra‐large (150%). We selected the color blue as previous studies examining color‐emotion association within interior built environments have found it is most neutral to participants (Güneş & Olguntürk, 2020), and due to the intrinsic properties of light and human visual system, blue is less frequently used for signage to affect behavioral action and/or inhibition responses (Braun & Silver, 1995). To achieve greater ecological validity of a color we might encounter in built settings, we matched the properties to a popular paint from a large local manufacturer. The experimental conditions are illustrated in Figure 1 and listed in Table 1.

**TABLE 1:** All scale scenes were rendered with a white finish (R255, G255, B255, hue [°] = 0, saturation [%] = 0, and brightness [%] = 100, and smoothness [%] = 50). The resting‐state scene was rendered black (R0, G0, B0, hue [°] = 0, saturation [%] = 0, brightness [%] = 0). Finally, the color condition presented last had wall surfaces were rendered in blue (R122, G155, B173, hue [°] = 198, saturation [%] = 35.3, brightness [%] = 67.8, and smoothness [%] = 50)

	Scale (%)	Color (RGB)	Hue (°)	Saturation (%)	Brightness (%)	Smoothness (%)
Resting‐state	N/A	R0, G0, B0	0	0	0	N/A
Control	100	R255, G255, B255	0	0	100	50
Small	75	R255, G255, B255	0	0	100	50
Large	125	R255, G255, B255	0	0	100	50
Extra large	150	R255, G255, B255	0	0	100	50
Color	100	R122, G155, B173	198	35.3	67.8	50

### 2.3 Apparatus

The CAVE consisted of three projector walls (3 m wide × 2.4 m high) and a floor (2.4 m wide × 3 m long), each with Barco Galaxy NW‐12 stereoscopic projectors. Projectors connected to a series of image generators consisting of Nvidia Quadro P6000 graphic cards, synced using Quadro Sync II cards at 120 Hz (60 Hz per eye) to render images at the same time. An optical‐based tracking system connected back to a Virtual Reality Peripheral Network (VRPN) Server (eight cameras, 240 Hz with submillimeter accuracy) tracked used movement through active LED markers located on the stereoscopic glasses. These were calibrated to ensure there was no conflict between eye height and postural cue (Leyrer et al., 2015). The VR environment was built using Unity with Vertical Sync (VSync) set to 60 frames per second.

To account for physiological comfort variables which could impact on the results, we measured and reported indoor environmental quality variables. Data were acquired through the CR100 Measurement and Control System with LoggerNet 4.6.2 software (Campbell Scientific, Inc.) at one‐minute intervals which were date and time stamped. We then averaged the one‐minute recordings of each session to create an average per participant session, and then average across all participant sessions. Variables recorded included interval sound pressure levels, overall mean air and wet‐bulb globe temperature, carbon dioxide concentration, and relative humidity. These were all within acceptable range for optimal performance as previously reported in Bower et al. (2022a) and Bower et al. (2022b).

### 2.4 EEG acquisition and analyses

EEG data were acquired using a 64‐channel EEG system (Philips Hydrocel Geodesic Sensor Net 64‐channel HCGSN) and recorded using Net Station 5 Geodesic EEG software, version 5.4.2 (Electrical Geodesics Inc.) using a sampling rate of 1000 Hz, with Cz as the online reference. We created a continuous recording with manual time stamps at the start and end of each two‐minute scene exposure using markers for each participant. We preprocessed the EEG data using EEGLab (v2019.1) (Delorme & Makeig, 2004), an open source graphic user interface and toolbox plugin for MATLAB R2019b (v9.7.0.1471314, MathWorks, Inc.). Data were band‐pass filtered (1–70 Hz; zero‐phase Butterworth filter) with a notch filter (47–53 Hz) to remove electrical interference from the CAVE environment. To aid the removal of recording noise, we applied the SOUND algorithm using input parameters of five iterations to evaluate noise in each channel and 0.2 regularization level (lambda value) to control the amount of cleaning (Mutanen et al., 2018). Each participant's continuous EEG data were decomposed using independent component analysis (FastICA algorithm) (Hyvärinen & Oja, 2000), with artifactual components identified with assistance from the ICLabel plugin (Pion‐Tonachini et al., 2019). We then extracted into conditions and segmented into three‐second epochs, resulting in 40 epochs per participant. Full details regarding EEG artifact correction and additional preprocessing are provided in the Extended Data Appendix (Supporting Information).

### 2.5 Functional connectivity

The EEG signal was subjected to a frequency transform using a single Hanning taper (1–70 Hz, frequency resolution of 1 Hz) to return the complex Fourier spectra for each subject/electrode across each EEG frequency band in 1 Hz steps: delta (1–3 Hz), theta (4–7 Hz), alpha (8–12 Hz), beta (13–30 Hz), low‐gamma (30–45 Hz), and high‐gamma (55–80 Hz). We compared phase‐based connectivity between all electrodes for each frequency band using the weighted phase lag index (wPLI) in FieldTrip toolbox for EEG/MEG‐analysis (Hardmeier et al., 2014; Oostenveld et al., 2011). wPLI was selected to minimize any effects related to volume conduction (Cohen, 2015; Plonsey & Heppner, 1967; Stinstra & Peters, 1998) (Figure 1).

### 2.6 Statistical analysis

The analysis was performed using the Network‐Based Statistic (NBS) Toolbox v1.2 (Zalesky et al., 2010; Zalesky et al., 2012) and MATLAB R2019b (v9.7.0.1471314, MathWorks, Inc.). The NBS approach is a method based on the graph model, which controls the family‐wise error rate when comparing pairwise relations between neural elements (Zalesky et al., 2010). The primary threshold (test‐statistic) was 3.2 for the control to scale (equivalent to *p* = .002), and 3.4 for color (equivalent to *p* = .003) conditions. The secondary significant threshold was set to *p* = .025 (two‐tailed) for family‐wise corrected cluster analysis (5000 permutations). BrainNet Viewer v1.7 (Xia et al., 2013) (http://www.nitrc.org/projects/bnv/) was used for visualizing the results. We also extracted the average connectivity strength across the significant subnetworks identified through NBS for each participant and condition. We then averaged these values to visually compare the difference in connectivity strength which we exported to RStudio (Version 1.3.959) for analysis and visualization.

## 3 RESULTS

### 3.1 Changes to built environment scale increased functional connectivity in the theta and high‐gamma bandwidths

**FIGURE 2:**
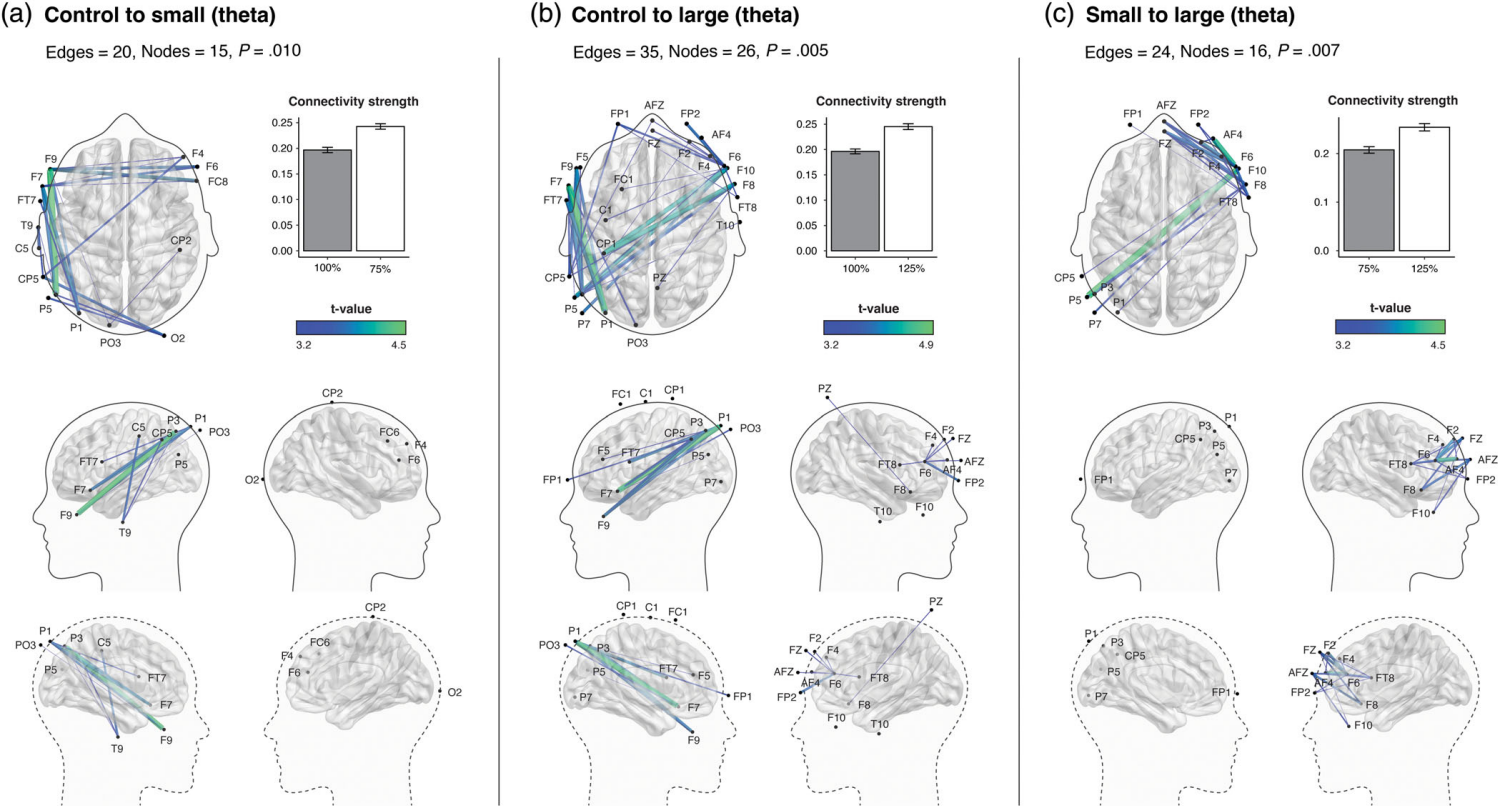
Effects of built environment scale on whole brain functional connectivity in the theta bandwidth. Images illustrate the functional subnetworks identified using the network‐based statistic (NBS). Group differences obtained using a t‐statistic of 3.2, which are depicted by the color gradient scale. Thickness of edges corresponds to functional connectivity strength (Pearson correlation). Clockwise top to bottom: Axial view, bar graphs depicting the average connectivity strength across all edges of the significant subnetworks (error bars denote SEM), lateral and medial views.

**FIGURE 3:**
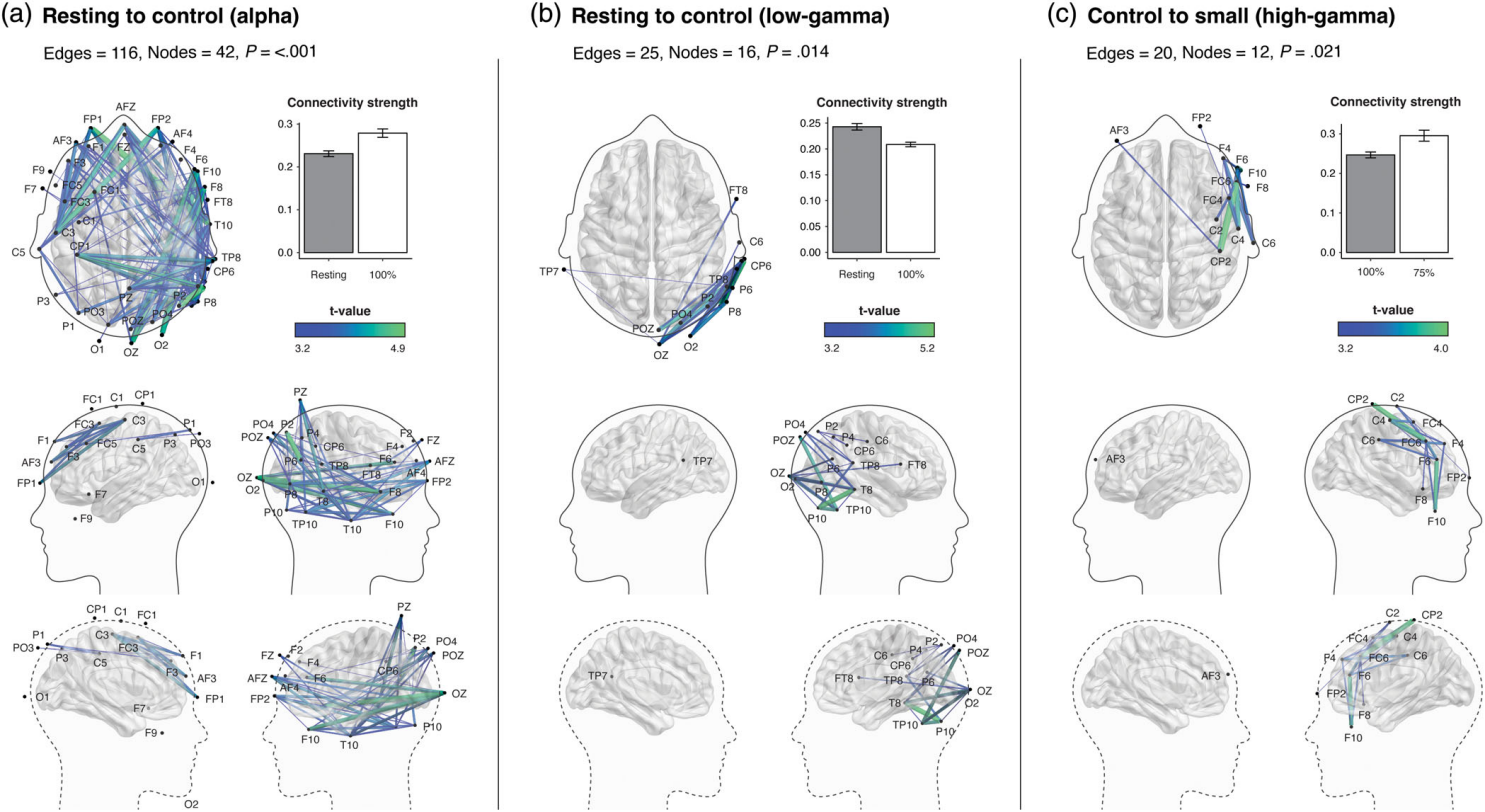
Effects of built environment scale on whole brain functional connectivity in the alpha and gamma bandwidths. Images illustrate the functional subnetworks identified using the network‐based statistic (NBS). Group differences obtained using a t‐statistic of 3.2, which are depicted by the color gradient scale. Thickness of edges corresponds to functional connectivity strength (Pearson correlation). Clockwise top to bottom: Axial view, bar graphs depicting the average connectivity strength across all edges of the significant subnetworks (error bars denote SEM), lateral and medial views.

Scale was found to modulate connectivity in the theta and high‐gamma bandwidths. Theta connectivity was increased when comparing the control to the small condition (20 edges, 15 nodes, *p* = .010). When comparing the control to the small condition, NBS identified a significant network of increased theta connectivity (20 edges, 15 nodes, *p* = .006). The identified network encompassed left temporal and parietal regions, as well as bilateral frontal regions. We also found a significant network of increased theta activity when comparing the control to the large condition (35 edges, 26 nodes, *p* = .005). This was asymmetrically spread across the brain, in the left hemisphere and across the midline from the left temporal to right frontal region. We also observed between‐condition effects in the theta range with increased connections when comparing the small to the large condition (24 edges, 16 nodes, *p* = .005). The network identified by NBS for small to large predominantly comprises frontal activity in the right hemisphere, connecting to the left temporal region (Figure 2). In the high‐gamma range, the NBS identified increased connectivity when comparing the control to small condition (20 edges, 12 nodes, *p* = .015). This activity was concentrated in the right frontoparietal region (Figure 3). However, we did not detect any significant connectivity within the remaining conditions.

### 3.2 Exposure to built environment control scale scene modulated functional connectivity in the alpha and low‐gamma activity

We also detected a significant subnetwork of connectivity in the alpha and low‐gamma bandwidths. However, this was confined to the eyes‐open resting‐state and control in both bands. Increased alpha connectivity from the resting to control (116 edges, 42 nodes, *p* = <.001) was widespread, comprising electrodes over frontal, central and posterior regions. Although widespread, there was significant clustered activity in the right temporo‐occipital region. In the low‐gamma range, the NBS identified increased connectivity networks when comparing the resting‐state to the control condition (25 edges, 16 nodes, *p* = .011). This was largely confined to the right occipital region. We did not detect any significant connectivity changes within the delta, beta, or low‐gamma frequencies (Figure 3).

### 3.3 Color (blue) increased functional connectivity in the theta bandwidth

**FIGURE 4:**
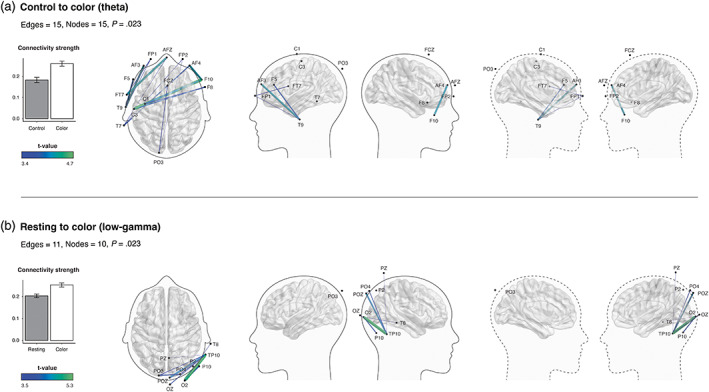
Effects of color on whole brain functional connectivity in the theta and gamma bandwidths. Group differences obtained using a t‐statistic of 3.4, which are depicted by the color gradient scale. Thickness of edges corresponding to functional connectivity strength (Pearson correlation). Left to right: Bar graphs depicting the average connectivity strength across all edges of the significant subnetworks (error bars denote SEM), axial view, lateral, and medial view.

We also found color modulated functional connectivity in the theta bandwidth. When comparing the control white scene with the blue condition, the NBS identified a significant network of increased theta connectivity (15 edges, 15 nodes, *p* = .023). This network consisted of interhemispheric activity across the frontoparietal region. We did not detect differences between the resting‐state and white control condition, suggesting the presentation of the achromatic built environment scene itself did not affect functional connectivity. The NBS also revealed a significant network of increased activity in the low‐gamma range when comparing the resting‐state to the color condition (11 edges, 10 nodes, *p* = .022). This was clustered in the right occipital region. No significant changes were detected in the remaining delta, alpha, or high‐gamma bandwidths (Figure 4).

## 4 DISCUSSION

This study interrogated whether the design characteristics of scale or color of interior built environments affect functional connectivity within the brain. Using a controlled approach through a CAVE, monitoring IEQ variables responsible for physiological comfort, and ensuring the scene was contextually neutral, we were able to detect differences in functional connectivity to smaller and larger built environment scale, and color in theta oscillations. We found that decreasing (control to small) and increasing (control to large, small to large) the scale of the built environment resulted in theta connectivity enhancement. We also detected increased high‐gamma connectivity comparing the control to small condition. Differences in alpha and low‐gamma connectivity were also found, but these were between the control and eyes‐open restingstate rather than scale conditions. When analyzing color, theta functional connectivity was also found to increase between the control (white) and color (blue) condition. Significant low‐gamma connectivity also increased from the resting‐state to the color condition. These findings are indicative of an association between exposure to different elements of the built environment and patterns of functional connectivity within the brain. Although further investigation is required to replicate this finding and understand the precise neural mechanisms involved, the application of these findings may have implications for several underlying processes such as cognitive, attentional, perceptual, and emotional functioning.

This initial study was interested in examining the neural correlates of changes to the scale and color of interior built environments, and future work will attempt extend these initial findings by exploring brain behavior relationships between the neural response and emotional, cognitive, and social behaviors. A key strength of this study is the use of the CAVE with EEG and IEQ monitoring to ensure ecological validity. Only a handful of studies worldwide have combined EEG measurement with an immersive CAVE environment (Bower et al., 2022a; Bower et al., 2022b; Edelstein et al., 2008; Pavone et al., 2016; Pezzetta et al., 2018; Vecchiato et al., 2015; Zappa et al., 2019), and our study is the first we are aware of which further controlled for IEQ variables and context within the space. Although debate exists around embodiment and the need for studies where participants move through spaces, we believe it is important to also understand the impact of being still within a space, as we spend a significant amount of time in prolonged stationary positions. That is, sitting in a school classroom or an office, or lying in a hospital bed. As a result of COVID‐19 protocols to reduce transmission, practices which had encouraged movement in the built environment such as shared desk and workspaces (encouraging workers to move locations) and using circulation spaces (stairwells and corridors) for dual functionality (additional workspace, etc.), has been discouraged. This has increased the exposure we have to a static environment, therefore, having an evidence base to elucidate differences or similarities between our responses when immersed in built environments in both stationary and mobile situations, is critical to our understanding.

Although this is a relatively nascent field of research, the findings of this study can be explored through the oscillatory bandwidths and brain connectivity patterns to interpret the underlying cognitive meaning of findings and the type of processes involved. However, with limited work conducted in this field, we caution that interpreting these findings remains speculative. As this study employed an eyes‐open resting‐state design, functional brain connectivity patterns consistent with activation of the default mode network, dorsal attention network, and visual processing networks would be expected. Attentional studies have suggested functional connectivity between the prefrontal and parietal cortex, are involved in visuospatial attention, with leftward hemispheric activity involved when attention is focused (Heinen et al., 2017). This pattern aligns with our results for reducing (control to small) and enlarging scale (control to large) in the theta bandwidth, suggesting the change in scale may have activated the visuospatial attentional network. Where the change in scale was greater in distance (small to large), we detected frontal right hemispheric connectivity. Activity lateralized to the right side of the brain including the temporoparietal junction and the ventral frontal cortex have also been found to interrupt cognitive activity when a stimulus of behavioral importance is detected (Corbetta & Shulman, 2002). This may explain the functional connectivity we found in the theta bandwidth when scale increased, as it may have triggered a need to adjust behavior of interaction with the substantially different scale.

Mismatch negativity, an electrophysiological response reflecting automatic detection of a violation of expectation in the sensory environment (Pazo‐Alvarez et al., 2003), can also offer a perspective for interpreting the results. Studies have also suggested frontal midline theta is involved in conflict responses, making it a potential candidate for communicating top‐down control across networks (Cavanagh & Frank, 2014; Nigbur et al., 2012). Here, our results could indicate the sensory stimulus input does not align with memories and prediction of an interior room, resulting in a conflict response.

Finally, it is important to understand the potential neurocognitive significance of the bandwidths we detected. Theta oscillations have broadly been linked to sensory information processing and transfer of information across brain regions (Colgin, 2013), as well as memory encoding and retrieval (Klimesch, 1996; Staudigl & Hanslmayr, 2013). Similarly, as gamma is proposed to be involved in perceptual feature binding, the results may reflect brain processes involved in memory formation, as part of a process to assist in future recollection (Burgess & Ali, 2002). However, given gamma activity is associated with undertaking complex cognitive tasks (Fitzgibbon et al., 2004), it is worth exploring in future studies whether the involvement of high‐frequency activity has an impact on task performance‐based measures.

Several limitations should be considered when interpreting the results of this study. In particular, the exposure time to the built environment conditions. We selected a two‐minute task free exposure to each of the scenes to limit habituation and fatigue. This may not reflect long‐term effects of built environment scale exposure as we spend a substantial amount of time inside buildings. Our experimental length also affected what we were able to test. Here, we opted not to extend the study length by also randomizing variations in the chair and door location. Future research could explore whether changing these locations under the same conditions elicits a different response. We also note the color variable of this study was limited to white and blue, future work could include other color levels. Next, there may be differences in resting‐state EEG functional connectivity versus performing task‐based activities, warranting further investigation. There are also spatial limitations in using EEG to determine functional connectivity. However, using fMRI, which provides excellent spatial resolution, significantly limits the ecological validity of the study, as participants are required to be horizontal and in a highly constrained spatial environment with a head cage. One possible compromise is to pursue magnetoencephalography (MEG) which could improve spatial resolution while also enabling the participant to have greater immersion through a virtual projection. The development of optically pumped magnetometer‐based MEG also has the potential to progress the field further (Seymour et al., 2021). Finally, this study used a CAVE as an environmentally controlled and cost‐effective simulation, enabling greater sensorimotor integration than VR headsets. Although early studies validate there is no significant difference in neurophysiological measures between real‐world and virtual‐environment stimuli (Kalantari et al., 2021), it is still important to ensure findings are replicable. Further research is required to go beyond examining the general effect of the environment to understand exactly how the exposure might interact with task‐based processes that reflect the real‐world dynamics of workplace activities, caregiving at home, and so forth. Similarly, it is important we unravel the relationships and effect of additional variables of the built environment (context, comfort, contents, occupants) through carefully disentangling and testing these in subsequent studies.

The results of this study provide exciting insights into how the brain is affected by our built environment surrounds, helping us to generate a research foundation where we can produce guidelines that enable a proactive building design approach for optimizing cognitive processes and supporting mental health. Recognizing the value and integrating neuroscience methods will be a pivotal step forward for the future of research and practice in the built environment discipline. We believe this research, as a component of the growing field of environmental psychology, needs to be a core component of built environment theory, education, and practice. Similarly, it is important for the field of neuroscience to recognize, report, and account for factors in the physical environment, as these may unwittingly alter experimental results. The next step to this research is determining what these connectivity changes reflect, and whether they might reflect conflict response (thus, potentially relating to health), or a cognitive response to the environment (with ramifications for education, healthcare, residential, commercial, and workplace settings). Crucially, debate is required to unravel the ethical implications and responsibilities of policy makers, planners, and building designers to ensure built environments are supportive and have a positive effect on mental health of the public. We believe it is important that we further interrogate the impact of building design on a broader subset of the population to further untangle whether built environment design is one of the underpinning elements of environmental enrichment which can serve as a neuroprotective factor for both healthy and clinical subsets of the population. This research will help us predict and evaluate the effect of the built environments we inhabit on brain and body functioning, so we can work towards designing buildings for optimal cognitive function and mental health.

## CONFLICT OF INTEREST

The authors declare no conflict of interests.

## Supporting information


**APPENDIX S1** Supporting Information

## Data Availability

The code and data that support the findings of this study are available in Open Science Framework (https://osf.io/9bsxe/?view_only=4f62dd37a189494a803e0d4d59f58a2b).
