# Supplementary material for: Functional brain connectivity during exposure to the scale and color of interior built environments
Source: Hum Brain Mapp. 2022 Sep 2;44(2):447–57. doi: 10.1002/hbm.26061 (PMC9842925; doi:10.1002/hbm.26061)
Supplement: Supplementary file 1 — APPENDIX S1 Supporting Information [file HBM-44-447-s001.docx]

**Supporting Information**

**EEG artifact correction and preprocessing**

We preprocessed the EEG data using EEGLab (v2019.1) (Delorme & Makeig, 2004), an open source graphic user interface and toolbox plugin for MATLAB R2019b (v9.7.0.1471314, MathWorks, Inc). We used a band-pass filter with a 1 to 70 Hz (zero-phase Butterworth filter) on continuous EEG data. A 47-53 Hz notch-filter was applied to exclude electrical interference from the CAVE environment. We then removed eye channels and the Cz reference channel. Next, we rejected channels if the kurtosis value was >5 standard deviations outside the average and replaced information in those channels using a spherical spline interpolation. Data were subsequently rereferenced to the average of all electrodes. To aid the removal of recording noise we applied the SOUND algorithm using input parameters of 5 iterations to evaluate noise in each channel and 0.2 regularization level (lambda value) to control the amount of cleaning (Mutanen et al., 2018). Each participants continuous EEG data were decomposed using independent component analysis (FastICA algorithm) (Hyvärinen & Oja, 2000), with artifactual components identified with assistance from the ICLabel plugin (Pion-Tonachini et al., 2019). A component was removed if ICLabel classified if the probability of that component containing brain data was less than 30% and the component was not in the ‘other’ category.

Using the time-stamped event markers in the continuous recording, each file was then split into 120 second block files using the start marker for each condition. Data were segmented into three-second epochs for subsequent analyses. Finally, additional artefact rejection was performed to remove any epochs with data exceeding ± 150 µV using the EEGLab ‘pop_eegthresh’ function. Lastly, we converted data from each participant/electrode to the frequency domain using the Fast Fourier Transform (FFT) with Hanning taper in the FieldTrip toolbox for EEG/MEG-analysis (1 Hz frequency steps between 1 to 70 Hz) (Oostenveld et al., 2011). After cleaning we calculated the average epochs remaining for each condition and participant (mean epoch = 39.5, ± 1.46).
